# *Salmonella enteritidis*: a rare pathogen in a polymicrobial acute prosthetic joint infection following total hip arthroplasty treated with double debridement, antibiotics, and implant retention (DAIR)

**DOI:** 10.1093/jscr/rjaf502

**Published:** 2026-02-08

**Authors:** Ahmad Hammad, Ahmad Tayim, Michael Daaboul, George Araj, Jean Paul Rizk, Bernard Sagherian

**Affiliations:** Department of Orthopedics Surgery, American University of Beirut Medical Center, PO Box 11-0236, Riad El Solh 1107-2020, Beirut, Lebanon; Department of Orthopedics Surgery, American University of Beirut Medical Center, PO Box 11-0236, Riad El Solh 1107-2020, Beirut, Lebanon; Department of Orthopedics Surgery, American University of Beirut Medical Center, PO Box 11-0236, Riad El Solh 1107-2020, Beirut, Lebanon; Department of Pathology and Laboratory Medicine, American University of Beirut Medical Center, PO Box 11-0236, Riad El Solh 1107-2020, Beirut, Lebanon; Department of Orthopedics Surgery, American University of Beirut Medical Center, PO Box 11-0236, Riad El Solh 1107-2020, Beirut, Lebanon; Department of Orthopedics Surgery, American University of Beirut Medical Center, PO Box 11-0236, Riad El Solh 1107-2020, Beirut, Lebanon

**Keywords:** salmonella, prosthetic joint infection, debridement, implant retention, total hip arthroplasty

## Abstract

Prosthetic joint infection (PJI) with *Salmonella enteritidis* is rare following orthopedic surgery and joint arthroplasty. This is a case of a 77-year-old female with left femoral transcervical fracture underwent total hip arthroplasty. At 2-weeks follow-up, the patient developed a PJI that was treated with double debridement, antibiotics, and implant retention (DAIR). Intraoperative cultures revealed unusual *S. enteritidis* without a primary focus of infection as part of polymicrobial PJI. She received a 3-month course of antibiotics including per os (PO) azithromycin and intravenous (IV) ceftriaxone that was switched to IV meropenem. At last follow-up, infection had resolved, inflammatory markers normalized, there was no evidence of prosthetic loosening and the patient was ambulatory. *Salmonella* organism is an atypical isolate in PJI. In the absence of a primary cause and in the context of acute infection, double DAIR is a viable option to salvage the prosthesis when a dual approach of aggressive intraoperative debridement and postoperative antibiotics coverage is employed.

## Introduction

Prosthetic joint infections (PJI) is a debilitating complication following total joint arthroplasty and is associated with increased morbidity, mortality, and reduced quality of life [1]. Biofilm formation on implanted prosthesis contributes to difficulty of infection eradication. *Staphylococcus* organisms are the most common, but unusual organisms are reported. *Salmonella* is a gram-negative organism that causes gastroenteritis; mortality is expected to double due to antimicrobial resistance [2, 3].

Treatment of PJI necessitates surgical intervention and antibiotic coverage for an extended period. PJI chronicity influences surgical approach. Two-stage revision remains the gold standard for chronic infections. Debridement, antibiotics, and implant retention (DAIR) has gained interest as an alternative option in acute infections; it is attractive due to limited iatrogenic bone loss, limited mobility restrictions, and high success rate [1, 4, 5].

To the best of our knowledge, there has been no previous report of polymicrobial PJI including *Salmonella enteritidis* in the absence of primary focus of infection, that has been treated with double DAIR operation.

## Case presentation

**Figure 1:**
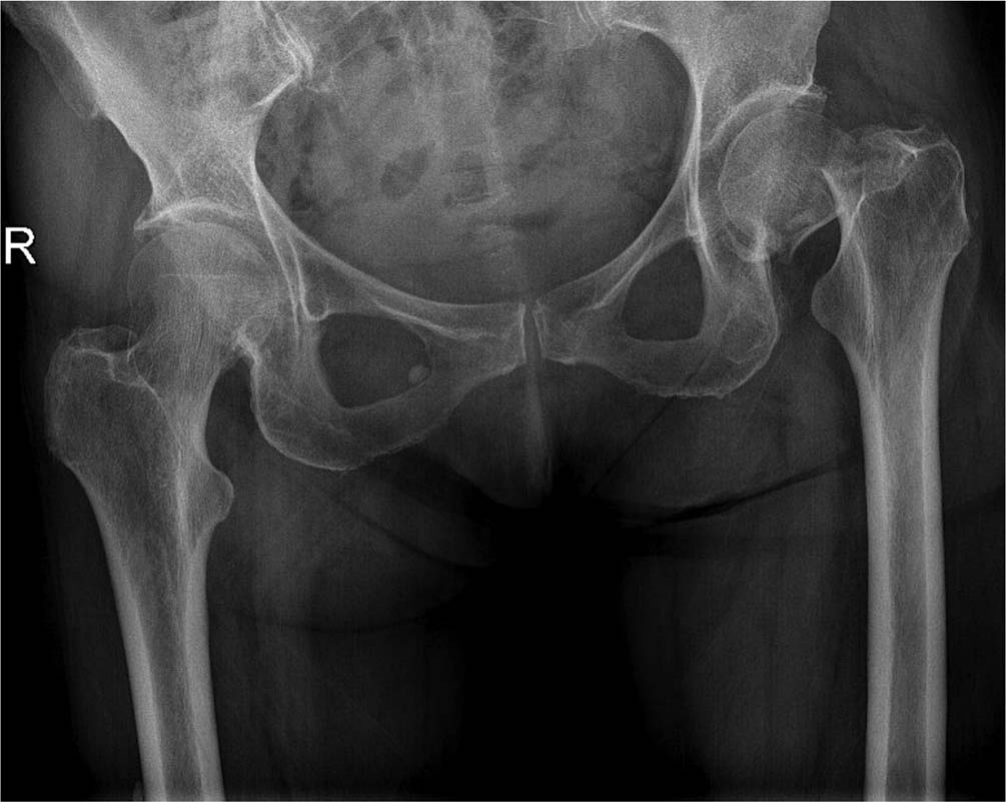
X-ray pelvis showing acute displaced and impacted left femoral transcervical fracture.

A 77-year-old female comorbid patient presented to the emergency department following a mechanical fall with acute displaced left femoral transcervical fracture (Fig. 1). Laboratory tests showed white blood cell count (WBC) count 11 800, C-reactive protein (CRP) 2.9, erythrocyte sedimentation rate (ESR) 18, and negative urine culture.

**Figure 2:**
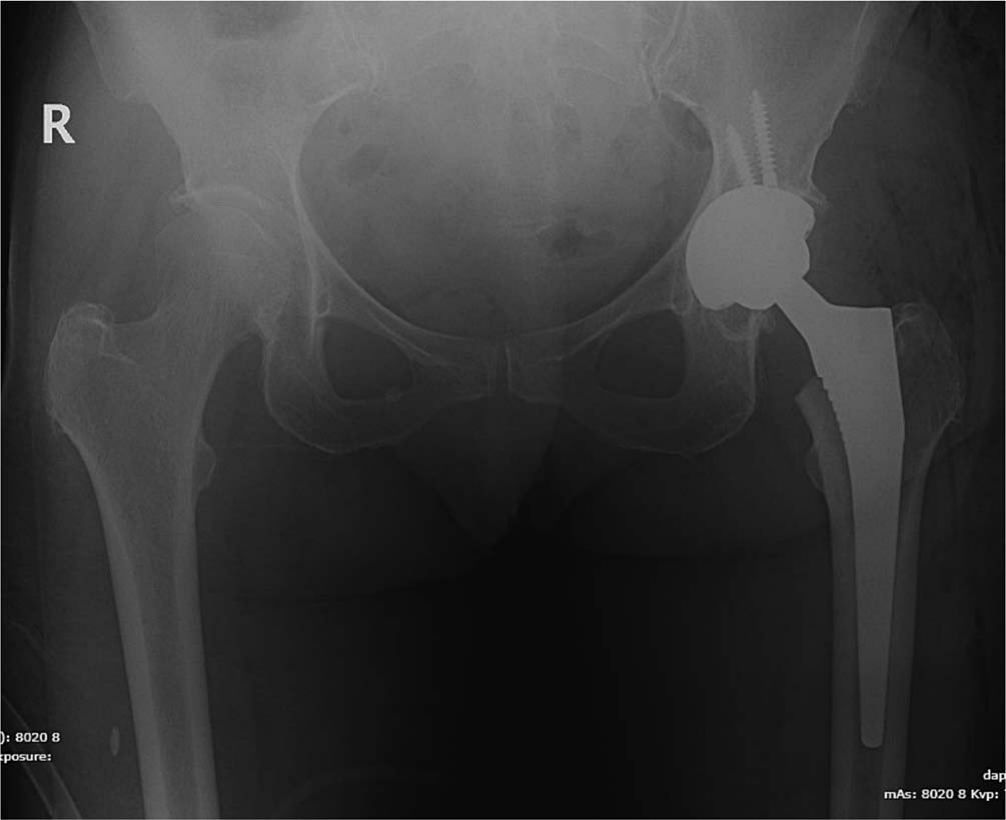
X-ray pelvis showing left total hip replacement with hardware in adequate position.

The patient underwent left total hip arthroplasty (Fig. 2). Her hospital stay was smooth and uneventful without any complications. Her wound was clean and dry without erythema or oozing and was discharged on prophylactic anticoagulation and pain medications.

**Figure 3:**
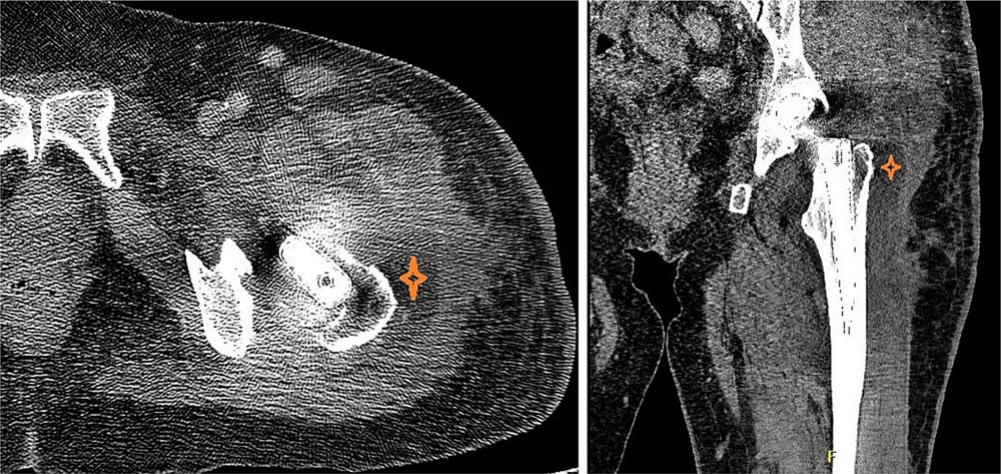
CT of the hip with contrast showed peripherally enhancing deep fluid collection (marked with star) containing gas pockets abutting the left proximal femur extending to the subcutaneous tissue and the gluteus medius muscles and measuring 7.2 × 6.6 × 14 cm with overlying skin thickening and fat stranding.

At the 2 weeks post-op visit for suture removal, she reported fever reaching 38.5°C for a few days with slight wound erythema. Blood tests showed WBC count 10 900 (83% neutrophils), ESR >120, CRP 310, Procalcitonin 1.24. Computed tomography (CT) scan of the hip with contrast (Fig. 3) showed deep fluid collection containing gas pockets abutting the left proximal femur, extending from subcutaneous tissue to gluteus medius and iliopsoas muscles, measuring 7.2 × 6.6 × 14 cm.

In the setting of an acute PJI, double debridement, antibiotics, and implant retention (double DAIR) and exchange of modular components were discussed to salvage the prosthesis. Intraoperatively, 20 cc of thick purulent fluid was extending from superficial to deep layers. Multiple tissue and fluid cultures were taken. The femoral head was removed, thoroughly brushed and kept in betadine solution. The acetabular liner was removed and discarded. Thorough synovectomy and debridement of infected tissues were performed. The modular component interfaces were extensively brushed and cleansed with betadine solution. The wound was soaked with betadine solution and then thoroughly irrigated with pulse lavage using 9 l of normal saline. Surgical gowns and gloves were then changed. New set of drapes was applied and a new set of instruments was used for the remainder of the procedure. The femoral head was washed and reapplied and a new acetabular liner was inserted. Antibiotic impregnated beads were prepared and applied over #1nylon suture, using 3 g of vancomycin in 40 g gentamicin impregnated cement. Two strings of beads were applied around the prosthesis. The patient was started on broad spectrum IV antibiotics, ceftazidime, and vancomycin.

Intra-op cultures grew *S. enteritidis*. To rule out any enteral focus, stool cultures collected twice and were negative. Blood cultures were negative, and urine culture showed *Enterococcus faecalis*. Antibiotics were switched to IV ceftriaxone and oral azithromycin for double coverage of *S. enteritidis*.

**Figure 4:**
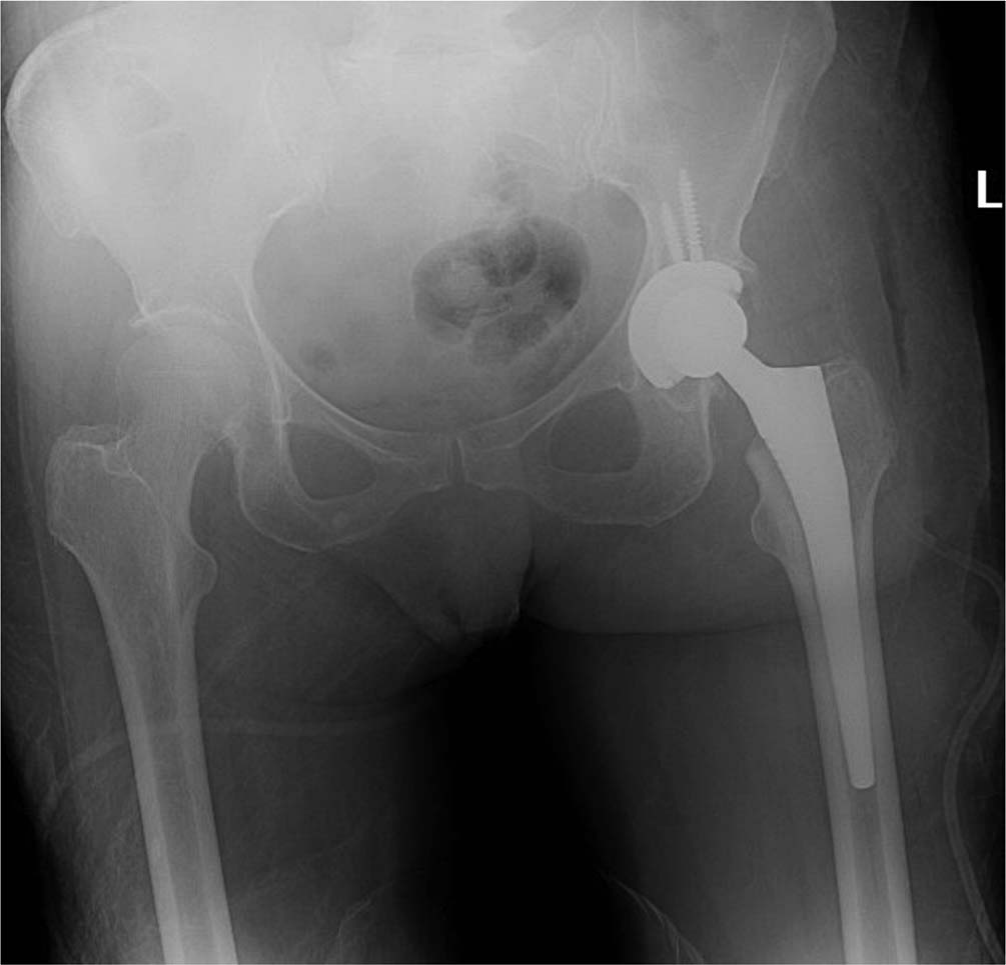
X-ray of the pelvis showing metallic hardware in good position.

Five days after the first DAIR procedure, the patient was taken to the operating room for the second DAIR and removal of previously applied antibiotic beads. No purulence was encountered and all tissues were healthy. The femoral head and acetabular liner were removed and discarded. Multiple tissue and fluid culture were again taken. Thorough brushing was done at the modular interfaces. The wound was soaked with betadine solution and again irrigated with pulse lavage using 9 l of normal saline. Gowns and gloves were changed by all surgical staff. Draping was done using sterile drapes over the previous drapes and a new set of surgical instruments was utilized for the remainder of the procedure. A new femoral head and acetabular liner were applied. Post-op imaging showed the hip prosthesis in adequate alignment (Fig. 4).

Intra-op cultures taken during the second DAIR grew *S. enteritidis* and *Klebsiella pneumoniae* (from thioglycolate broth).

**Figure 5:**
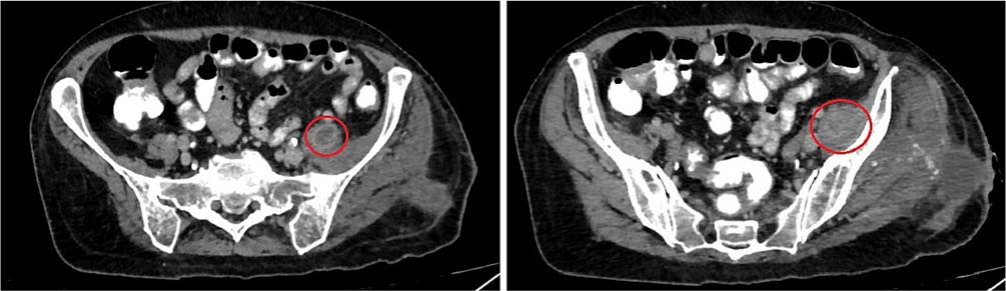
CT abdomen/pelvis showing rim enhancing fluid collection (marked with circle) in the left psoas muscle (left image) with extension to the left iliacus (right image) measuring 8.7 × 2 × 1.5 cm, suggestive of abscess formation.

**Figure 6:**
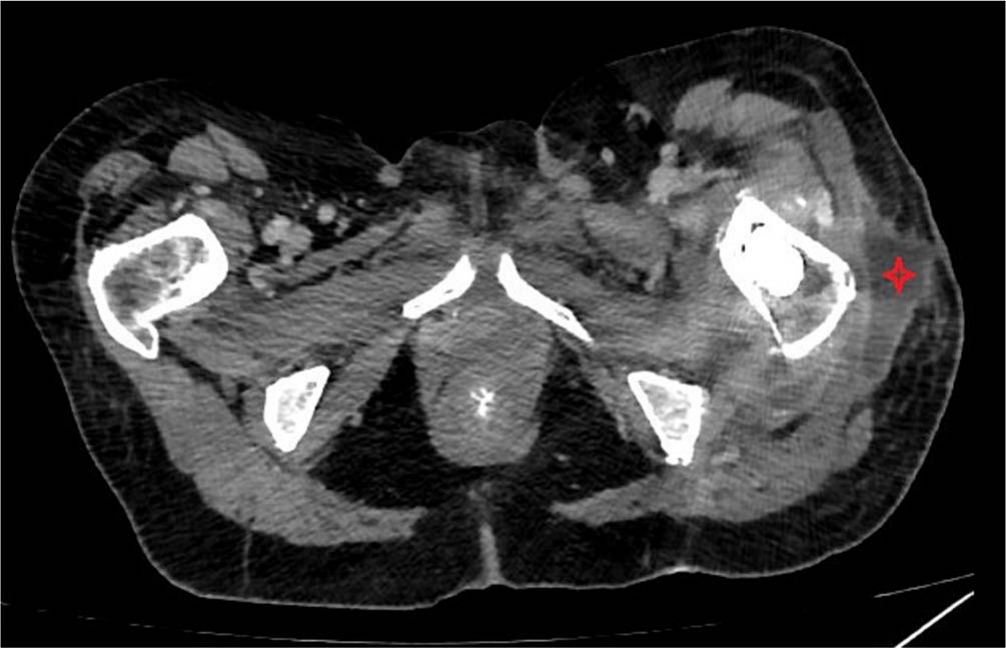
CT abdomen/pelvis showing subcutaneous collection at the upper lateral aspect of the thigh in the vicinity of the left proximal femur.

One week after the second stage DAIR, lab tests showed WBC 7500 (63% neutrophils), ESR 102, and CRP 48. CT abdomen and pelvis with IV contrast at 2 weeks did not show any intraabdominal collection or fistula suspicious of primary source of seeding, however it showed fluid collection in the left psoas muscle extending to the iliacus (8.7 × 2 × 1.5 cm), suggestive of deep abscess formation (Fig. 5) with superficial collection in vicinity of left proximal femur (Fig. 6). The latter collection was superficial and was drained at the bedside, releasing non-purulent fluid. The deep abscesses were drained by interventional radiology and a pigtail catheter was inserted for 1 week. Cultures from the deep collection grew *K. pneumoniae* susceptible to meropenem, which was used to replace ceftriaxone.

The patient was discharged home to continue a total of 12 weeks of IV meropenem and PO azithromycin. On follow-up visits, her inflammatory markers decreased. Follow-up CT scan showed resolution of the psoas abscess. At last follow-up, more than 8 months after the double DAIR procedure, her CRP was 4.3 and she was ambulatory without any aids.

## Discussion

The incidence of PJI after hip arthroplasty is 0.5%–2%; acute PJI occurs within 3–6 weeks of surgery. Chronic PJI occurs later and is characterized by biofilm formation. Proper intraoperative mechanical debridement and postoperative antibiotics is necessary for optimal biofilm eradication and infection control [4].

*Salmonella* is a rare cause of bone and joint infections and atypical in PJI. Few reports exist on *Salmonella* pathogenesis following knee and hip arthroplasties, with *Salmonella typhimurium* and *enteritidis* being most commonly identified [6]. Non-surgical treatment of PJI is associated with high failure rates [7]. In acute PJI, DAIR has become an attractive option that prevents unnecessary removal of stable prosthetic implants, thus reducing the risk of bone stock loss and fracture [1].

The success rate of DAIR in acute PJI reaches 71% [5]. When treated within 6 weeks of index surgery, 68% of acute PJI are successfully eradicated with the first DAIR which increases to 85% in multiple DAIR [1]. The latter is associated with good functional outcomes and 77% 10-year implant survivorship. The reduced success rate is secondary to prolonged interval (>30 days), immunocompromised state and mismatch between antibiotics and pathogen susceptibility. Early PJI diagnosis and treatment, exchanging modular components, adequate mechanical debridement and proper duration of targeted antibiotics, increase infection eradication and improve prosthetic survival [1, 4, 5].

Modular component exchange, head and polyethylene liner, remains controversial. The main purpose of components exchange is to access areas under the trunnion and liner for adequate debridement of potential remnants. It is associated with decreased reoperation (28% vs 44%) and higher prosthesis survival (71.4% vs 55.5%) [8]. Complications include persistence of infection (15%) and dislocation (14%) [1]. Failure of DAIR necessitates a two-stage revision. The outcome of subsequent two-stage revision for PJI is not compromised after a failed DAIR [9]. There is identical success rate between isolated two-stage revision and revision following failed DAIR (82.2% vs 82.5%) [10].

This case of PJI by *Salmonella* spp. might be attributed to a nosocomial polymicrobial infection given the absence of a primary focus of infection. The treatment of polymicrobial PJI is challenging and lengthy, especially in the presence of unusual pathogens. Timely diagnosis, surgical debridement, and targeted antibiotics remain key for infection eradication. Further research is needed to compare the efficacy of DAIR compared to two stage revision in acute PJI secondary to atypical pathogens.
